# A classification framework for exploiting sparse multi-variate temporal features with application to adverse drug event detection in medical records

**DOI:** 10.1186/s12911-018-0717-4

**Published:** 2019-01-10

**Authors:** Francesco Bagattini, Isak Karlsson, Jonathan Rebane, Panagiotis Papapetrou

**Affiliations:** 1 Dipartimento di Ingegneria dell’Informazione, University of Florence, Florence, Italy; 2 Department of Computer and Systems Sciences, Stockholm University, Stockholm, Sweden

**Keywords:** Electronic health records, Adverse drug events, Data mining, Sparse multi-variate features, Temporal abstraction, Machine learning, Shapelets

## Abstract

**Background:**

Adverse drug events (ADEs) as well as other preventable adverse events in the hospital setting incur a yearly monetary cost of approximately $3.5 billion, in the United States alone. Therefore, it is of paramount importance to reduce the impact and prevalence of ADEs within the healthcare sector, not only since it will result in reducing human suffering, but also as a means to substantially reduce economical strains on the healthcare system. One approach to mitigate this problem is to employ predictive models. While existing methods have been focusing on the exploitation of static features, limited attention has been given to temporal features.

**Methods:**

In this paper, we present a novel classification framework for detecting ADEs in complex Electronic health records (EHRs) by exploiting the temporality and sparsity of the underlying features. The proposed framework consists of three phases for transforming sparse and multi-variate time series features into a single-valued feature representation, which can then be used by any classifier. Moreover, we propose and evaluate three different strategies for leveraging feature sparsity by incorporating it into the new representation.

**Results:**

A large-scale evaluation on 15 ADE datasets extracted from a real-world EHR system shows that the proposed framework achieves significantly improved predictive performance compared to state-of-the-art. Moreover, our framework can reveal features that are clinically consistent with medical findings on ADE detection.

**Conclusions:**

Our study and experimental findings demonstrate that temporal multi-variate features of variable length and with high sparsity can be effectively utilized to predict ADEs from EHRs. Two key advantages of our framework are that it is method agnostic, i.e., versatile, and of low computational cost, i.e., fast; hence providing an important building block for future exploitation within the domain of machine learning from EHRs.

**Electronic supplementary material:**

The online version of this article (10.1186/s12911-018-0717-4) contains supplementary material, which is available to authorized users.

## Background

Although electronic health records (EHRs) have been extensively exploited for developing robust predictive models and for solving challenging predictive modeling tasks in healthcare [1, 2], EHRs still present critical problems that need to be solved so as to fully exploit the complex interactions and information they contain. Recent studies estimate that in the United States adverse drug events (ADEs), and other preventable adverse reactions in the hospital setting, incur, apart from the human suffering, a yearly monetary cost of approximately $3.5 billion [3]. Therefore, it is of paramount importance to reduce the impact and prevalence of ADEs within the healthcare sector, not only since it will result in reducing human suffering, but also since it can substantially reduce the economical strains on the healthcare system. Although benefit–risk analysis of newly developed drugs is already conducted during clinical trials, post-marketing detection and surveillance are necessary to detect unanticipated events. Clinical trials are normally performed with a limited sample of patients, who are followed for a limited period of time. As a result, not all serious adverse events can be detected prior to market deployment, which results in drugs being withdrawn due to serious adverse reactions not detected during clinical trials. To overcome some of these limitations, several attempts have been made to manually encode rules for detecting ADEs in EHRs [4–6]. However, in addition to requiring substantial efforts by domain experts to formulate such rules, the objectives typically change over time which requires the manually encoded rules to be frequently updated. More importantly, however, many ADEs are not identified, due to the limited knowledge about effects of medical treatments, such as drugs being tested only in limited trials and under controlled conditions.

Hence, an alternative approach towards ADE detection is to resort to machine learning for exploiting the constantly growing volume of EHR data, and more specifically to effectively exploit the inherently complex nature of these data sources. Indeed, a lack of investigation in utilizing EHR predictive modeling for ADEs built on structured medical data (e.g, laboratory test results) has led to interest in the development of machine learning models, such as random forests, which can aid in altering the clinical courses of ADE vulnerable patients [7]. The development and application of predictive models in a clinical setting can result in substantial improvements when it comes to ADE detection while minimizing the inherent costs.

The adoption of EHRs has increased the interest towards secondary use of clinical and medical data by researchers and practitioners [8, 9]. Examples of the obvious and detrimental benefits of EHR systems include public health surveillance, pharmacovigilance, healthcare quality assessment and monitoring [10]. Moreover, the employment of EHRs facilitates opportunities for ADE investigations to move from individual-level to population-level research, a facet which has broadly received increased attention within clinical and translational research [8].

The vast majority of research on learning from EHRs has been focusing mainly on four key categories [11]: (1) comorbidity detection and analysis, (2) patient clustering, (3) predictive modeling, and (4) cohort analysis and querying. Specific examples of such categories include: association rule mining, classification or prediction of patient conditions such as identification of the smoking status of patients [12], patient safety and automated surveillance of adverse events [13], comorbidity and disease networks [6], processing of clinical text [14], identification of suitable individuals for clinical trials [15], and the identification of temporal associations between medical events and first prescriptions of medicines for signaling the presence of an ADE [16].

### Temporal abstractions of EHRs

Additional complexity is also induced on the EHR feature space from time series variables which can often be of different lengths, measured at irregular time intervals, or exhibit high levels of sparsity. Several attempts have been made to address this problem, and retrofit standard machine learning methods for building predictive models from such feature spaces. One family of studies resort to handling temporal variables by considering simpler mappings, i.e., converting each time series feature value to a static representation, also known as *temporal abstraction*. Examples of such simple mappings include the length, average, mean, slope, or the weighted sum of all values of the time series [17–20]. Although the mapping heuristics allow standard predictive modeling techniques to be employed directly, they compromise the quality of the feature space by nullifying the underlying temporal information of the variables; information which may be immensely useful for the predictive task at hand [21]. As shown in earlier studies and data domains where variables are time series, and characterized by high missing-value rates, the best performing temporal abstraction has been to consider counts of the values in each variable.

An alternative approach is to employ off-the-shelf time series summarization techniques, where the goal is to reduce the length of the time series by transforming them into more compact representations without loss of information and by preserving the notion of temporal order. Examples of such summarization techniques are, among others, the piecewise aggregate approximation (PAA) [22] and its follow-up version called symbolic aggregate approximation (SAX) [23, 24], the discrete Fourier transformation (DFT) [25] in the frequency domain, and the discrete wavelet transformation (DWT) [26]. Among those representations, SAX, maps the time series to a symbolic sequence; in particular, this mapping is achieved by assigning each continuous value to a symbol from a discrete alphabet that follows from a Gaussian distribution. More importantly, SAX is oftentimes preferred against other representations due to its simplicity [24].

Moreover, a basic concept that has been used extensively for feature-based time series classification is that of time series *shapelets*, which typically refer to class-distinctive time series subsequences [27–29]. In a typical time series classification problem, an object is described either by only a single (univariate) or a set (multi-variate) of time series of equal length, that are measured at equal time intervals. The multi-variate time series case could be considered as an equivalent formulation to our problem, as each feature could be seen as a single “channel” of a multi-variate time series. Nonetheless, our setup differs substantially as in our case: (1) each individual time series is not necessarily sampled at fixed time intervals and (2) our multi-variate features are highly sparse, i.e., they contain many missing values.

Approaches focusing on classification of epidemiological longitudinal data [30, 31] handle multi-variate feature variables by employing what is known as *population-based* feature extraction. Population-based methods are, however, limited to very short time series variables of up to three measurements, and are not suited for long and sparse multi-variate features. In fact, the closest approach and direct competitor of our work is the *random dynamic subsquence* method proposed by Zhao et al. [32], where the main idea is to convert the temporal features to SAX sequences, choose a representative subsequence for each feature, and compute the distance of all features to the representative. This process results in single-valued features. Unfortunately, the approach by Zhao et al. [32] suffers from two main weaknesses: (1) the chosen distance function, i.e., Levenshtein distance, is highly dependent on the sequence length and (2) it cannot effectively handle and exploit the high degree of sparsity in the feature space.

### ADE knowledge extraction from EHRs

Previous work automatic detection of ADEs from EHRs has consisted of a variety of approaches such as discovering statistical links between certain ADEs and drug dosage [33] alongside natural language processing using unstructured data such as clinical notes [34, 35]. However, there remains a deficiency for investigating ADEs with predictive modelling on structured EHR data. Existing predictive modelling studies have utilised standard techniques such as regression for large-scale mining of ADEs [36] and random forest classifiers applied to clinical codes and measurements [37]. However, what most studies lack is the exploitation of crucial information regarding the temporal order of clinical events which may have a profound impact on the classification performance of particular ADEs. One such approach was proposed with the objective of detecting ADE signals focusing on laboratory abnormalities after treatment with specific medication [38]. The formulation of our paper is rather different, as in our case we are using all clinical measurement signals concurrently to learn temporal features for building an ADE classifier. These features can be of any type, normal or abnormal, and they are used by the classifier as long as they constitute good ADE predictors.

Recent work by Zhao et al. [32] has incorporated temporal information through the use of symbolic sequence representations of EHR time series data for the purpose of ADE detection. In this study we seek to improve upon the work of Zhao et al. by accounting for and exploiting high levels of feature sparsity inherent to EHR data.

The main focus of our paper is proposing a predictive modeling based framework that utilizes multi-variate temporal features, allowing traditional machine learning algorithms to work for complex and time-evolving data sources. EHRs contain such complex data, but predictive modeling methods typically rely on data sources to be in a structured form, i.e., a tabular format, where objects (in our case patient records) correspond to rows and attributes (in our case patient variables) correspond to columns [39]. Nonetheless, EHR data can rarely fit into such format due to its inherent complexity, induced, for example, by the prevalence of longitudinal observations. A clinical variable for a particular patient, for instance, is not always described by a single value, but by a series of values over time. Consequently, the induced data table may contain features for which their data type is a time series variable, instead of a real or categorical variable.

### Contributions

Our work focuses on the use of EHR data for the application area of ADE detection, as it constitutes a serious and ubiquitous public health issue. Unfortunately, most approaches to ADE detection in EHRs do not take into account the temporality of clinical events, which is critical for this task, while they cannot effectively handle sparsity in the feature space since in a medical context values are not missing at random (MNAR) [40–42]. The technical contributions of this paper are, thus, summarized as follows:

- we propose a sparse symbolic representation for multi-variate feature spaces, with emphasis on temporal features of arbitrary lengths and high degree of sparsity, i.e., missing values. The proposed representation is based on the SAX time series summarization technique as well as on the concept of *s-shapelets*, which correspond to class-distinctive discrete event subsequences;
- we propose three strategies for dealing with such feature spaces: (1) length encoding or plain (which is an extension of Zhao et al. [32]), most-common encoding or mc, and left-right optimized encoding or lr;
- we provide an extensive experimental evaluation of the three strategies on 15 real datasets taken from the healthcare domain involving ADEs. Moreover, we study the utility of the chosen s-shapelets, as well as their consistency to medical findings in the context of ADE detection.

## Methods

### Overview

Our hypothesis is that sparse and unevenly sampled feature variables from EHRs can serve as strong predictors of an ADE. More concretely, given an EHR dataset represented in the form of unevenly sampled and sparse multi-variate features, our goal is to infer a classification model that is able to correctly predict the presence of an ADE for a previously unseen medical record.

**Fig. 1:**
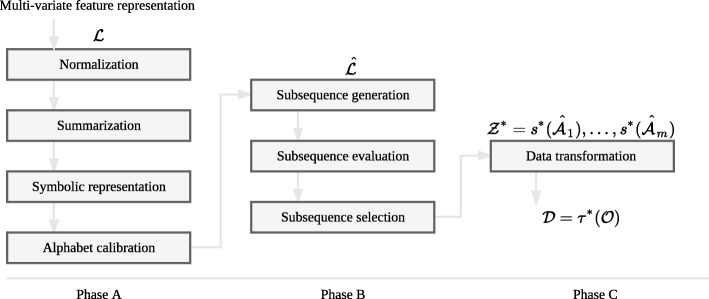
The framework. A graphical representation of the transformation framework
- **Phase B:** a set of candidate subsequences is generated from the discretized features, which are then evaluated based on their class-distinctive power (referred to as *utility*), and finally the set of subsequences with the highest utility, called *s-shapelets*, is generated. An important component of this phase concerns the way empty data records, i.e., records with empty sequences, are processed. Towards this end we propose three strategies for handling and exploiting empty sequence features.
- **Phase C:** using the s-shapelets extracted from the training set, each multi-variate data feature (of the training and test sets) is converted to a real-valued feature.

As depicted in Fig. 1, our proposed framework consists of the following *three phases*:

- **Phase A:** each feature in the training set is first transformed into a discrete symbolic sequence representation.

### Definitions and problem formulation

We now provide some basic definitions and the formulation of the problem at hand.

#### **Definition 1**

A time series *S*={*s*_1_,…,*s*_*d*_} is an ordered set of *d* real values, where each $s_{k} \in \mathbb {R}$, with |*S*|=*d*.

The classification task involves a set of data features, where each feature is represented by a time series. We denote such features *multi-variate features*.

#### **Definition 2**

A multi-variate feature space $\mathcal {A} =\{A_{1}, \ldots, A_{m}\}$ is a set of *m* multi-variate features, where each $A_{j} \in \mathcal {A}$ is a time series.

Using this multi-variate feature space $\mathcal {A}$ we can define a *multi-variate object*$O \in \mathcal {A}$ as an instantiation of that feature space. Moreover, given a set of predefined class labels $\mathcal {Y}=\{y_{1}, \ldots, y_{\sigma }\}$, we can define the universe of *labeled multi-variate objects* in that space. Due to the particular prediction task, the experiments only considers binary labels, *σ*=2. However, the proposed strategy handles multiple labels, i.e., *σ*>2 without modification.

#### **Definition 3**

Given a multi-variate feature space $\mathcal {A}$ and a set of predefined class labels $\mathcal {Y}$, the *universe of labeled multi-variate objects* is defined as a set of tuples $\mathcal {O}=\{(O_{i},y_{i})~|~O_{i}\in \mathcal {A}, y_{i}\in \mathcal {Y}\}$, where *y*_*i*_ is the class label assigned to object *O*_*i*_, $\forall (O_{i}, y_{i})\in \mathcal {O}$, with *i*∈[ 1,*n*].

In a typical classification setting, we are given a set of training objects, where each object is associated with a class label, called the *training set*. In our case, we employ a *multi-variate training set*, denoted as $\mathcal {L}$, which is simply drawn from our universe of labeled multi-variate objects $\mathcal {O}$. In other words, $\mathcal {L}\subseteq \mathcal {O}$.

Our task is to learn a classification function *f* for mapping the objects in a multi-variate training set $\mathcal {L}$, defined as instantiations of the multi-variate feature space $\mathcal {A}$, to the set of class labels $\mathcal {Y}$. In other words, we want to learn a mapping $f : \mathcal {A} \rightarrow \mathcal {Y}$, such that for each $O_{i}\in \mathcal {L}$

$$f(O_{i}) = \hat{y}_{i}\in \mathcal{Y} \, \forall i\in \{1,\dots,n\} \, $$

where $\hat {y}_{i}$ denotes the predicted class label for object *O*_*i*_.

Using the above definitions, the problem studied in this paper is defined as follows:

#### **Problem 1**

Given a universe of labeled multi-variate data objects $\mathcal {O}$ defined over a multi-variate feature space $\mathcal {A}$, a multi-variate training set $\mathcal {L}$, and a loss function *Δ*, the objective of *multi-variate feature classification* is to learn a mapping function $f: \mathcal {A} \rightarrow \mathcal {Y}$ using $\mathcal {L}$, such that the classification error on (unseen) labeled data objects drawn from universe $\mathcal {O}$ is minimized. The classification error is expressed by the expectation of the loss function:

$$E_{(O_{i},y_{i})\in\mathcal{O}}[\!\Delta (y,f(O_{i}))] \ . $$

In this work, we will consider the 0/1 loss function:

$$\Delta (y,y^{\prime}) = \left\{ \begin{array}{ll} 0,& \text{if} y = \hat{y}\\ 1, & \text{otherwise} \end{array} \right. $$

In the following three subsections we describe each of the three phases of the proposed framework in detail.

### Phase A: Multi-variate feature discretization

The objective of the first phase is to discretize the space of multi-variate features $\mathcal {A}$, resulting into a new feature space, where feature values correspond to symbolic sequences. We denote this target space $\mathcal {\hat {A}}$ and refer to it as *multi-variate symbolic feature space*. The discretization process follows four steps: (1) normalization, (2) summarization, (3) symbolic representation, and (4) alphabet calibration. Since the multi-variate features are practically instantiated as time series variables, we employ standard time series normalization, summarization, and symbolic representation techniques for the following steps. Nonetheless, in principle one could use any alternative technique for each of the four steps.

#### Normalization

Each multi-variate feature variable is first z-normalized, i.e., the observed mean is subtracted by each value while also dividing by the observed standard deviation. In other words:

1 $$ S := \frac{{\sum\nolimits}_{i=1}^{|S|}\{s_{i}-\mu(S)\}}{\sigma(S)} \,,  $$

where *μ*(*S*) and *σ*(*S*) correspond to the mean and standard deviation of the values of *S*.

#### Summarization

Next, the multi-variate features are converted to their corresponding PAA representations [43]. Given a fixed parameter *w*, a time series *S* of length *d* can be mapped to a *w*-length representation $\overline {S} = \{\overline {s}_{1}, \ldots, \overline {s}_{w}\}$, where the *i*^*th*^ value of $\overline {S}$ is computed as follows:

2 $$ \overline{s}_{i} = \frac{w}{d} \sum\limits_{j=\frac{d}{w}(j-1)+1}^{\frac{d}{w}i} s_{j} \ .  $$

Hence, PAA results in a length reduction from *d* to *w*, by splitting *S* into *w* partitions of equal size, and assigning each partition the mean value of the points of the original time series falling into that partition.

#### Symbolic representation

Next, each value of $\bar {S}$ is mapped to a discrete symbol defined over an alphabet *Σ* of size *α*. For this symbolic representation we employ a standard time series summarization technique called SAX [23, 24].

More concretely, a mapping is defined between $\mathbb {R}$ and an alphabet *Σ* of *α* symbols. One assumption is that each time series variable is generated by an underlying distribution, e.g., a Gaussian. Next, a set of *α*−1 breakpoints $\mathcal {B} =\{\beta _{1}, \ldots, \beta _{\alpha -1}\}$ are defined, so that the area under the Gaussian normal curve N(0,1) from each pair (*β*_*i*_, *β*_*i*+1_) is equal to 1/*α*, assuming that *β*_0_=−*∞* and *β*_*α*_=*∞*. Hence, given a desired alphabet size *α*, the breakpoints can easily be defined by consulting a statistical table. Once the breakpoints are obtained, $\bar {S}$ is mapped to a sequence of symbols $\hat {S}$ as follows: all coefficients that are lower than the first breakpoint are mapped to the first alphabet symbol, e.g., *a*; the next set of coefficients with values between the first and the second breakpoints are mapped to the second available symbol, e.g., *b*; and so on. The resulting symbolic representation $\hat {S}$ is called the SAX approximation of *S*, defined over a SAX alphabet *Σ* of *α* symbols. By applying SAX, the initial multi-variate feature space $\mathcal {A}$ is converted to its SAX representation, which defines the symbolic multi-variate feature space $\mathcal {\hat {A}} = \left \{\hat {A}_{1}, \ldots, \hat {A}_{m}\right \}$, comprising symbolic sequence representations of variable lengths.

#### Alphabet calibration

As it can be seen, a parameter to calibrate when using SAX is the *alphabet size* *α*, which corresponds to the number of symbols that are used for mapping the normalized time series values. Ideally, a minimum number of symbols is needed to reflect the underlying dynamics of a time series. Since the latter is typically unknown, the choice of a proper alphabet size is mostly an empirical task [44].

At the end of Phase A, each multi-variate object *O* is converted to its symbolic counterpart, denoted as $\hat {O}$. Hence, the universe of multi-variate objects $\mathcal {O}$ is converted to a universe of labeled symbolic objects, denoted as $\mathcal {\hat {O}}$, and the converted training set is now denoted as $\mathcal {\hat {L}}$, while empty records in a data object are represented as the empty set (*∅*). Note that the instantiation in Phase A replicates the method described by Zhao et al. [32] to allow for assessing the gain of using the subsequent phases.

### Phase B: sub-sequence enumeration

In the second phase, after the original multi-variate feature space has been transformed to its symbolic representation, a pool of candidate representative subsequences is generated, evaluated, and the subset of most representative ones is finally selected. The overall objective of this phase is to identify a class-distinctive subsequence, which we denote as s-shapelet, for each multi-variate feature. This process, which follows three steps, is detailed below.

#### Subsequence generation

Assume we are given a symbolic sequence $\hat {S}$ of length $\left |\hat {S}\right |$, corresponding to an instantiation of feature $\hat {A}_{i}$, which is the symbolic representation of the original multi-variate feature *A*_*i*_. A subsequence *s* of $\hat {S}$ is defined as a sampling of length |*s*| of contiguous symbols from $\hat {S}$, such that $|s|\leq \left |\hat {S}\right |$, i.e., $s = \left \{\hat {S}_{t}, \ldots, \hat {S}_{t+|s|-1}\right \}$, with $|s|\leq t \leq -\left |\hat {S}\right |+1$.

Given the symbolic representation $\mathcal {\hat {L}}$ of a multi-variate training set $\mathcal {L}$, and an alphabet size *α*, we generate a pool of candidate subsequences, denoted as $\mathcal {S}_{\alpha }$, by randomly sampling the sequences in $\mathcal {\hat {L}}$. Practically, $\mathcal {S}_{\alpha }$ contains snippets of existing symbolic sequences in $\mathcal {\hat {L}}$ of arbitrary lengths in [1, *l*_*max*_], where *l*_*max*_ is the length of the longest sequence in $\mathcal {\hat {L}}$.

#### Sub-sequence evaluation

The set of randomly generated subsequences $\mathcal {S}_{\alpha }$ is next evaluated based on the *utility* of each sub-sequence. In our setting, the utility of a subsequence corresponds to its capability of splitting a training set into two disjoint partitions that separate the class distribution into pure subsets. More concretely, given a dissimilarity measure, *D*(·,·), between two discrete event sequences of the same length, a target sequence $\mathcal {\hat {S}}$ and another sequence *s*, with $|s| \leq \left |\mathcal {\hat {S}}\right |$, the distance function *Dist*(·,·) between *s* and $\mathcal {\hat {S}}$ is defined as follows:

3 $$  Dist\left(s, \mathcal{\hat{S}}\right) := \min_{s^{\prime}\subseteq \mathcal{\hat{S}}, \left|s^{\prime}\right| = |s|} \left\{ D\left(s, s^{\prime}\right) \right\}.  $$

Intuitively, the above distance corresponds to the dissimilarity between *s* and its best matching subsequence in $\mathcal {\hat {S}}$. Although *D*(·,·) can be any distance function for string matching, in this paper we use the *edit distance* [45], as it is one of the most widely used measures for evaluating string similarity.

We should note that in the approach described in Zhao et al. [32], referred to as *random dynamic subsequence*, a similar idea was used for measuring the distance between a candidate sub-sequence and a data sequence. The key difference in our paper is that we employ a modified edit distance function (Eq. 3), which computes the best *subsequence match* of candidate *s* in the target sequence, as opposed to a full sequence match computed by *random dynamic subsequence*. This is a substantial improvement of the competitor method as in its original version the used distance function is highly affected by the length of the target sequence; especially when $|s| << \left |\mathcal {\hat {S}}\right |$, the distance value becomes meaningless. In our case, the solution we propose (Eq. 3) makes the distance function invariant to the length difference of the two compared sequences.

Now, consider a training set $\mathcal {\hat {L}}$, as converted by phase A, and assume that it consists of *k* class labels. Moreover, let *p*(*y*_*i*_) be the proportion of sequences belonging to class *y*_*i*_, *i*∈[1,*k*], the *entropy* of $\mathcal {\hat {L}}$ can be defined as:

4 $$ I\left(\mathcal{\hat{L}}\right) := -\sum\limits_{i=1}^{k} p(y_{i}) log(p(y_{i})).  $$

Furthermore, if we partition $\mathcal {\hat {L}}$ into *q* disjoint subsets $\left \{\mathcal {\hat {L}}_{1}, \ldots, \mathcal {\hat {L}}_{q}\right \}$, the total entropy of the partitioning can be computed as:

5 $$ I\left(\left\{\mathcal{\hat{L}}_{1}, \ldots, \mathcal{\hat{L}}_{q}\right\}\right) := \sum\limits_{i=1}^{q} \frac{\left|\mathcal{\hat{L}}_{i}\right|}{\left|\mathcal{\hat{L}}\right|} I\left(\mathcal{\hat{L}}_{i}\right).  $$

Given the definition of entropy, the we can define the *information gain* a particular partitioning strategy yields on a dataset $\mathcal {\hat {L}}$, as:

6 $$ Gain\left(\left\{\mathcal{\hat{L}}_{1}, \ldots, \mathcal{\hat{L}}_{q}\right\}\right) := I\left(\mathcal{\hat{L}}\right) - I\left(\left\{\mathcal{\hat{L}}_{1}, \ldots, \mathcal{\hat{L}}_{q}\right\}\right).  $$

The utility of a sub-sequence in our pool of candidates $\mathcal {S}_{\alpha }$ is computed following the approach by Ye et al. [46]. More precisely, for each subsequence $s \in \mathcal {S}_{\alpha }$, we compute the dissimilarity between *s* and all the sequences in $\mathcal {\hat {L}}$, using function *Dist*(·,·) (Eq. 3) to induce a partitioning of $\mathcal {\hat {L}}$ into two disjoint subsets $\mathcal {\hat {L}}_{1}$ and $\mathcal {\hat {L}}_{2}$. For simplicity, we consider two-way splits, i.e., *q*=2, but the approach is generalizable to any number of partitions. Consequently the information gain given by *s* is evaluated (using Eq. 6) and measures the ability of *s* to separate $\mathcal {\hat {L}}$ into two partitions with class distributions of low entropy.

Finally, let $\mathcal {\hat {S}}$ denote a symbolic sequence of an object’s feature in $\mathcal {\hat {L}}$. To maximize the information gain, we seek for a distance threshold *δ*, such that each $\mathcal {\hat {S}} \in \mathcal {\hat {L}}$ is assigned to $\mathcal {\hat {L}}_{1}$ if $Dist(s, \mathcal {\hat {S}}) < \delta $ or to $\mathcal {\hat {L}}_{2}$, otherwise. Next, we extend the previous equation to define the gain achieved by a subsequence *s* with a given distance threshold *δ* as follows:

7 $$\begin{array}{*{20}l} Gain\left(s, \delta, \mathcal{\hat{L}}\right) := Gain\left(\left\{\mathcal{\hat{L}}_{1},\mathcal{\hat{L}}_{2} \right\}\right), \end{array} $$

where $\mathcal {\hat {L}}_{1} = \left \{\mathcal {\hat {S}} \! \in \! \mathcal {\hat {L}}:Dist\left (s, \mathcal {\hat {S}}\right) \! < \! \delta \right \}$ and $\mathcal {\hat {L}}_{2}=\left \{\mathcal {\hat {S}} \! \in \! \mathcal {\hat {L}}:Dist\left (s, \mathcal {\hat {S}}\right) \! \geq \! \delta \right \}$. In particular, we are looking for the value of *δ* inducing a split of $\mathcal {\hat {L}}$ with the lowest possible entropy. More concretely, inspired by the definition of *optimal split point* given in Ye et al. [46], we define such distance threshold as:

8 $$ \delta_{osp}\left(s, \mathcal{\hat{L}}\right) := \arg \max_{\delta} Gain\left(s, \delta, \mathcal{\hat{L}}\right).  $$

**Fig. 2:**
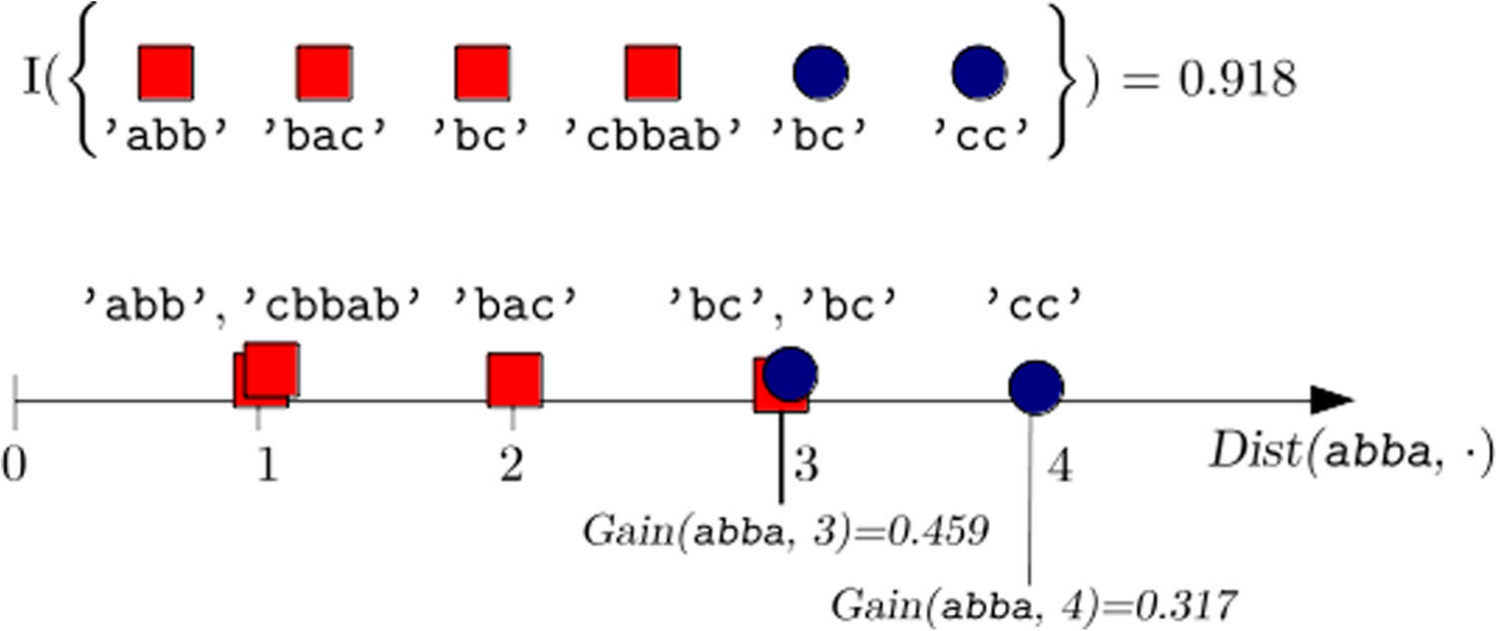
Simple subsequence evaluation. A graphical representation of how the subsequence *s*=’abba’ is evaluated and the corresponding optimal distance threshold *δ*_*osp*_(abba) is selected. On the top of the figure, a training set $\hat {\mathcal {L}}$ is represented as a collection of negative (red squares) and positive (blue circles) sequences; within each class, sequences are arranged in alphabetical order. The total label entropy of $\hat {\mathcal {L}}$ is equal to $I\left (\hat {\mathcal {L}}\right) = 0.918$. On the bottom, the sequences in $\mathcal {D}$ are arranged on an horizontal axis based on their distance *Dist*(abba,·) from the subsequence. Among all of the possible candidate thresholds *δ*∈[1,4], the two best are reported in the figure, namely *δ*=3 and *δ*=4, yielding an information gain of 0.459 and 0.317, respectively: therefore, *δ*_*osp*_(abba):=3 is chosen as the optimal splitting distance for the subsequence ’abba’


Algorithm 1 sketches the evaluation process described above, while Fig. 2 depicts a graphical example.

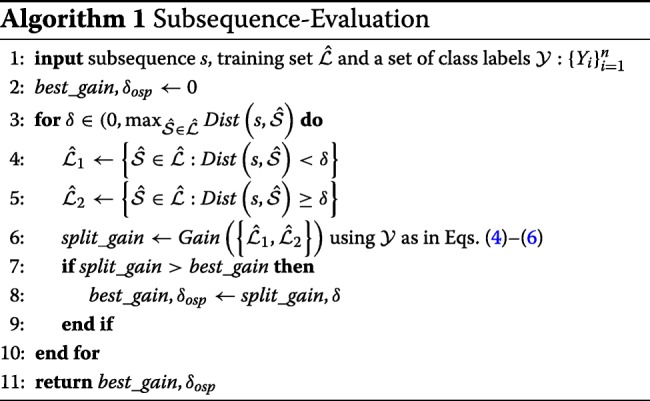


Assuming that our multi-variate feature space is sparse, i.e., a large fraction of the feature space in our raw dataset contains time series of zero length, represented as *∅*, missing data plays a crucial role when the optimal distance threshold is computed. In “Exploiting sparsity” section, we will explain how missing entries should be treated in this phase, and propose alternative methods to achieve this goal.

#### Sub-sequence selection

So far, we have transformed raw multi-variate features into a symbolic sequence dataset and used the latter to generate a set of candidate representative subsequences. We have also defined a way of ranking such candidates according to their utility, i.e., their ability to separate the dataset into two partitions with class distributions of low entropy.

Now, we want to identify the representative sub-sequence with the highest utility per multi-variate feature variable. We call this sub-sequence a *sequence shapelet* or *s-shapelet*.

##### **Definition 4**

Given a training set $\mathcal {L}$ and an alphabet *Σ*of size *α*, a *sequence shapelet* or *s-shapelet* *s*^∗^ is a discrete event sequence defined over *Σ*, which induces the partition of $\mathcal {\hat {L}}$ with the highest information gain, i.e.,

9 $$ s^{*} := \arg \max_{s \in \mathcal{\hat{L}}} Gain\left(s, {\delta}_{osp}(s), \mathcal{\hat{L}}\right) \,.  $$

Since an exhaustive search in the sub-sequence space can easily become infeasible [46, 47], the s-shapelet, within our framework, is selected from the (finite) set $\mathcal {S}_{\alpha }$. Hence, for a given $\mathcal {S}_{\alpha }$ defined over an alphabet *Σ* of a chosen size *α*, an s-shapelet $s^{*}_{\alpha }$ is defined as

10 $$ s^{*}_{\alpha} := \arg \max_{s \in \mathcal{S_{\alpha}}} Gain\left(s, {\delta}_{osp}(s), \mathcal{\hat{L}}\right) \,.  $$

The alphabet size for which the achieved information gain is maximized, denoted as *α*^∗^, is called *the alphabet size of maximum utility*, and is defined as follows:

11 $$ \alpha^{*} := \arg \max_{\alpha \in \mathcal{I}} Gain\left(s^{*}_{\alpha}, {\delta}_{osp}(s^{*}_{\alpha}), \mathcal{\hat{L}}\right)  $$

where $\mathcal {I}\subseteq \mathbb {N}$, i.e., is the set of candidate alphabet sizes.

Finally, the overall best s-shapelet *s*^∗^, which we call an *optimum s-shapelet*, corresponding to the shapelet that yields the maximum gain among all possible candidates of each alphabet size, is defined as follows:

12 $$ s^{*} := s^{*}_{\alpha^{*}}.  $$

In summary, for each feature $\mathcal {\hat {A}}_{j} \in \mathcal {\hat {A}}$ in the training set $\mathcal {\hat {L}}$, the optimum s-shapelet, $s^{*}\left ({\mathcal {\hat {A}}_{j}}\right)$, is selected which in fact is the s-shapelet with the highest utility for $\mathcal {\hat {A}}_{j}$ across all possible alphabet sizes in $\mathcal {I}$. The final product of this phase is the set of *m* optimum s-shapelets, one per feature in $\mathcal {\hat {A}}$, which we denote as $\mathcal {Z}^{*}=\left \{s^{*}\left ({\mathcal {\hat {A}}_{1}}\right), \ldots, s^{*}\left ({\mathcal {\hat {A}}_{m}}\right)\right \}$.

The only missing part of this phase is how we deal with feature sparsity, i.e., feature values corresponding to empty sequences. In “Exploiting sparsity” section, we will describe three strategies for dealing with sparse multi-variate features.

### Phase C: data transformation

The overall process including phases A and B can be summarized by a function $\tau (\mathcal {L}, w, \mathcal {I},\Sigma, \alpha, \emptyset)$, which takes as input all parameters of phases A and B, and finally results in a learned function *τ*^∗^(·), where all parameters are optimized as described in these two phases. Once *τ*^∗^(·) has been learned, it can be used to transform any data object of the original multi-variate space to a set of real-valued features. In other words, function *τ*^∗^(·) is simply a mapping, such that

13 $$ \tau^{*} : \mathcal{A} \rightarrow \mathbb{R}^{m} \,.  $$

Hence, any multi-variate object $O\in \mathcal {A}$ can be converted to a real-valued feature object, denoted as $\tilde {O}$, by applying function *τ*^∗^(·) to its original representation, i.e., $\tilde {O} = \tau ^{*}(O)$.

In practice, this transformation is performed by computing the distance (Eq. 3) between each $s^{*} \in \mathcal {Z}^{*}$ and its corresponding symbolic object feature. This transformation is performed to both the training set $\mathcal {\hat {L}}$, during the model training phase, as well as to the test set at prediction time. Intuitively, our data objects are transformed from a set of multi-variate features (columns), with each feature being a time-series variable, to a set of single-valued features, where each feature value corresponds to the distance between its symbolic representation to the selected optimum s-shapelet for that feature.

Finally, we should stress that, again, object instances with empty records require special attention. To this end, several design choices are needed in order to investigate whether or not to consider these empty records and how to represent them. These choices are described next.

### Exploiting sparsity

Throughout the transformation framework, mainly in Phase B, described in the previous sections, several design choices need to be made for handling and exploiting the sparsity of the feature space, i.e., data entries that are missing not at random. In this section, we highlight the steps where such choices are critical for exploiting these missing values towards improving prediction performance. To this end, we propose three strategies, which we call length encoding (or plain), most-common encoding (or mc), and left-right optimized encoding (or lr).

#### Strategy I: Length encoding (plain)

An encoding for missing entries is first needed when raw time series are mapped into SAX sequences, which. as mentioned earlier, are marked with *∅*. Based on Algorithm 1, when a candidate subsequence *s* is evaluated, an optimal distance threshold is determined in order to compute the information gain achieved by the data split induced by *s*. At this step, a decision has to be taken on whether or not and how to consider empty sequences (i.e., empty feature records) when computing the optimal threshold. For example, suppose we have a very sparse multi-variate training set $\mathcal {L}$, which, after its conversion to $\mathcal {\hat {L}}$, is mapped to a feature space of symbolic sequences with many empty strings (i.e., having a large fraction of *∅*).

A simple strategy is to apply Algorithm 1 directly (see lines (4)–(6)), so that the distance between *s* and *∅* will be simply equal to the length of the candidate subsequence, that is, *Dist*(*s*,*∅*)=|*s*|. As a result, all empty feature records will be assigned either to $\mathcal {\hat {L}}_{1}$ or $\mathcal {\hat {L}}_{2}$ based solely on the length of *s*. This strategy is referred to as *length-encoding* or plain.

In summary, plain treats empty multi-variate feature records as regular entries, and replaces them with the distance between their symbolic representation and the optimum s-shapelet, that is, it replaces them with $\left |s^{*}\left (\mathcal {\hat {A}}_{j}\right)\right |$ for each feature $\mathcal {\hat {A}}_{j}$. This approach is a modified and improved version of *random dynamic subsequence* used in Zhao et al. [32]. In particular, our method corrects for bias introduced by *random dynamic subsequences*, i.e., to favor longer subsequences, using our re-defined subsequence distance measure. As a consequence, we use plain as a baseline for methods that consider the temporal information, similar to how sl acts as a baseline for methods that does *not* consider temporal information in our experimental evaluation.

#### Strategy II: most-common encoding (mc)

An alternative strategy is to ignore empty feature records corresponding to empty sequences, and compute the optimal distance split by using only non-empty feature records. We call this strategy *most common* encoding or mc. In fact, when building the single-valued features at training and prediction time, mc replaces *∅*s with the distance value *Dist*(*s*^∗^,·) that occurs most frequently within the training set. When dealing with very sparse feature spaces, this choice can be interpreted as a way of considering missing entries as “frequent”.

Consider, for example, the feature space corresponding to clinical measurements of patients taken over different time periods. If a clinical measurement has been recorded only for a relatively small number of patients, in the corresponding dataset empty feature records will be ubiquitous. Thus, associating a missing entry with the most frequently (or commonly) observed value will mark it as a recurring event. Of course, this strategy is not guaranteed to work for dense feature sets, while replacing empty sequences with the most common distance may not capture the actual meaning of a missing measure.

#### Strategy III: Left-right optimized encoding (lr)

To overcome the limitations of both simple length-encoding and most-common value encoding, we introduce a third strategy, which we call *left-right* encoding or lr. When evaluating a distance threshold for a given subsequence and building the resulting split $\left \{\mathcal {\hat {L}}_{1},\mathcal {\hat {L}}_{2}\right \}$ (lines (4) and (5) of Algorithm 1), lr tries to assign all of the *∅*s either to $\mathcal {\hat {L}}_{1}$ (*left*) or to $\mathcal {\hat {L}}_{2}$ (*right*), and selects the option yielding the highest information gain. According to this choice, the distance of a candidate s-shapelet to *∅* is computed as follows:

14 $$  Dist(s, \emptyset) := \left\{ \begin{array}{ll} 0 & \emptyset \rightarrow \mathcal{\hat{L}}_{1} \\ \max\limits_{\hat{\mathcal{S}} \in \mathcal{\hat{L}}} Dist\left(s, \hat{\mathcal{S}}\right) & \emptyset \rightarrow \mathcal{\hat{L}}_{2}\\ \end{array} \right.  $$

**Fig. 3:**
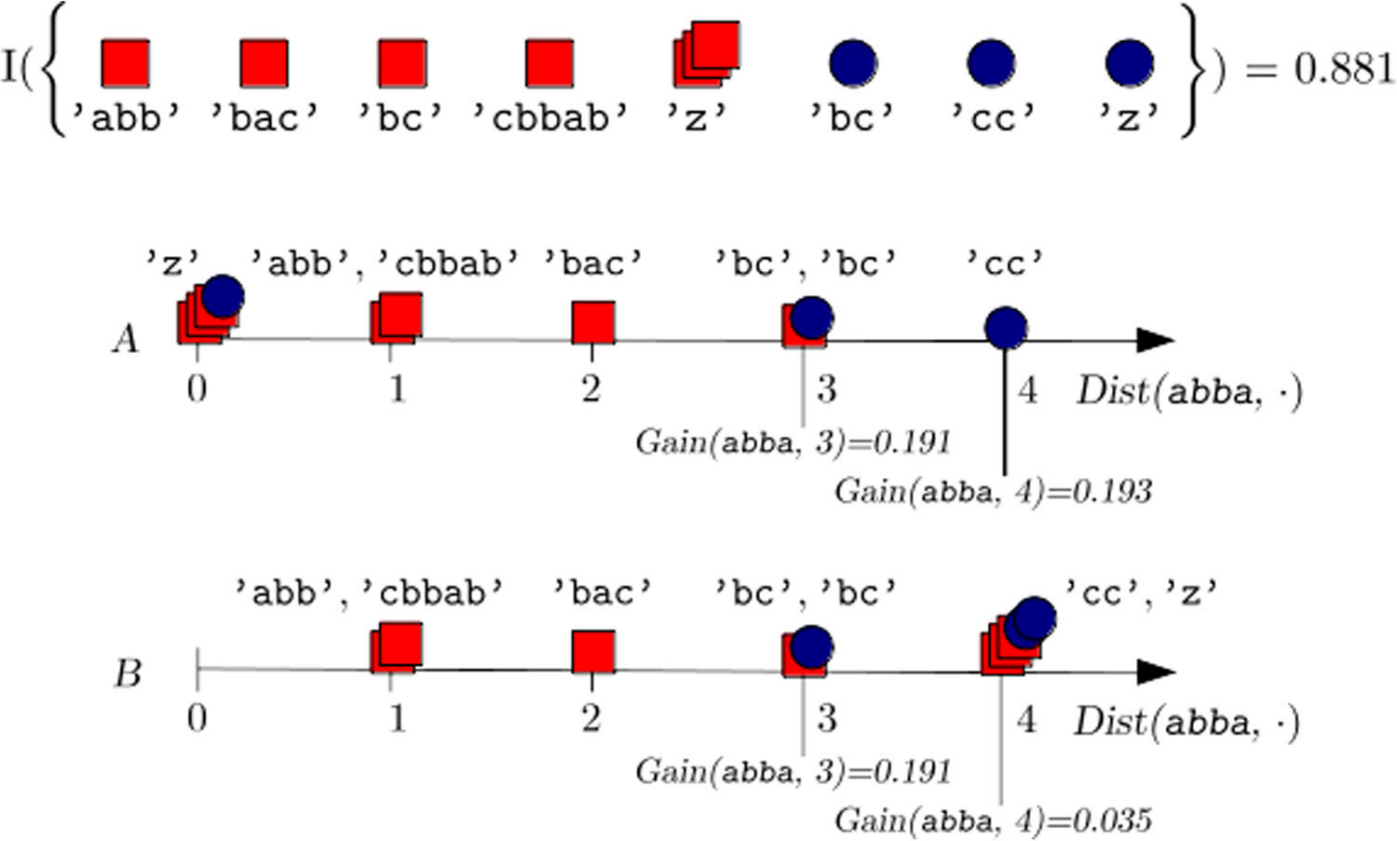
Advanced subsequence evaluation. A graphical example of how lr works. The scenario is similar to that of Fig. 2: on the top, a labeled sequence dataset $\hat {\mathcal {L}}$ is depicted, which contains both actual and empty sequences; the latter are marked by *∅*, while the total entropy of $\mathcal {\hat {L}}$ is equal to 0.881. Below the dataset representation, the sequences are arranged on the horizontal line reporting their distance from ’abba’: lr takes into account two cases, respectively marked with *A* and *B*. In *A*, lr places all *∅*s to the left side of the split, by assigning *Dist*(abba,*∅*):=0; conversely, in *B*, empty sequences are placed to the right side, with a distance from the shapelet equal to $Dist(\texttt {abba},\emptyset) := \max _{\mathcal {S}_{\alpha } \in \hat {\mathcal {L}}} = 4$. The optimal distance threshold is chosen based on the highest split gain among those obtained in *A* and *B*. The figure reports, for both cases, two threshold examples, namely *δ*=3 and *δ*=4: in particular, *δ*=4 turns out to be the best option, due to the gain (0.193) yielded in *A*. Therefore, *δ*_*osp*_(abba):=4

This additional computation is performed when deciding on the splitting distance value and does not affect the remaining transformation steps. Figure 3 provides a concrete example of the way in which lr selects the best s-shapelet. The above strategy also keeps track of the assignments that yield the overall maximum gain, that is, $Dist(s^{*}(\mathcal {A}_{j}), \emptyset)$, *j*=1,…,*m*, and uses this value to replace missing entries at both training and prediction time.

In “Utility of s-shapelets selected by lr vs. plain” section, we elaborate on the lr strategy in terms of interpretability and show that a dynamic encoding of missing data can (also) help understand the s-shapelet that has been selected for classifying a multi-variate feature dataset.

### Data source

The experiments carried out in this work are based on HealthBank [1, 48], which is an EHR database containing de-identified health records for approximately 1.2 million patients admitted to a hospital or local care facility in the Stockholm County region. The data was collected during 2009 to 2015 by Karolinska University Hospital. The data source contains a total of 11,623 unique diagnoses codes defined by ICD10-SE codes (The 10th revision of the International Statistical Classification of Diseases and Related Health Problems).

ADEs are reported using codes from the seven ADE categories proposed by Stausberg and Hasford [49]. Among these, A.1 and A.2 – *a drug-related* and *a drug- or other substance-related causation was noted in the diagnosis code*, respectively – indicate a clear sign of an ADE occurrence and are, hence, included as possible datasets in this study. Note that our data source does not contain a sufficient number of patient covering all of the ADE categories mentioned by Stausberg and Hasford [49].

### Empirical evaluation

We formulate our experiments as binary classification tasks. Hence, for each ADE we create a dataset where data examples correspond to patients that either have or have not been assigned with the corresponding ADE diagnosis code. In each dataset, positive examples are those patients who have been diagnosed with a specific ADE, while negative examples are those patients who have been given a diagnosis code that belongs to the same disease taxonomy, i.e., sharing the same first three levels of the ICD-10 hierarchy, but is not an ADE. For example, if the ADE under consideration is I95.2 (*Drug-induced hypotension*), patients who share the same code up to the third position are considered as negative examples, i.e., I95.* (*Hypotension*) (where * denotes any character) except for *I95.2*. In the experiments, we include inpatient encounter, but only predict the possibility of an ADE for their *last* encounter.

#### Datasets

We have selected all ADEs belonging to A.1 or A.2 [49] in our EHR data source (see Table 1), which have also been assigned to at least 50 patients by practicing physicians at the respective hospital and clinical department. Patients are described by the set of clinical laboratory test measurements, encoded using the NPU system [50] (see Additional file 1 for information about the least sparse codes), recorded for up to 90 days before the occurrence of each particular ADE, but not including the time point when the target ADE is assigned. In fact, we expect that using a time window of 90 days, and not include all past events, is more informative since recent events are likely to influence the target more than older events. Investigating the effect of the window size parameter and its optimal value is outside the scope of this study, however, it is not expected to have any major impact on the relative performance of the investigated approaches.

**Table 1:** Positive denotes patients which are ADE positive, whereas Negative corresponds to patients that are ADE negative

Adverse drug event	Positive	Negative
*D61.1*: **Aplastic anaemia**	593	105
Average age	50.3	55.6
Gender distribution (% female)	42.7	49.5
*E27.3* **Adrenocortical insufficiency**	70	259
Average age	61.8	56
Gender distribution (% female)	58.6	58.6
*G62.0* **Polyneuropathy**	96	783
Average age	59.8	72.7
Gender distribution (% female)	47.9	40.1
*I95.2* **Hypotension**	115	1287
Average age	79.3	74.7
Gender distribution (% female)	40.9	49.6
*L27.0* **Generalized skin eruption**	182	468
Average age	60.1	48.7
Gender distribution (% female)	55.5	48.4
*L27.1* **Localized skin eruption**	151	498
Average age	59.8	55.9
Gender distribution (% female)	50.3	54.5
*M80.4* **Osteoporosis**	52	1170
Average age	65.8	70.9
Gender distribution (% female)	71.15	81
*O35.5* **Damage to fetus by drugs**	146	260
Average age	38.5	38.9
Gender distribution (% female)	100	100
*T78.2* **Anaphylactic shock**	131	856
Average age	50.9	45.46
Gender distribution (% female)	50.4	60.7
*T78.3* **Angioneurotic oedema**	283	720
Average age	56.4	42.35
Gender distribution (% female)	59	59.9
*T78.4* **Allergy**	574	415
Average age	41.2	52.5
Gender distribution (% female)	65.2	51.2
*T80.1* **Vascular complications**	66	609
Average age	66.2	63.2
Gender distribution (% female)	48.5	64.7
*T80.8* **Infusion complications**	538	138
Average age	64.3	60.4
Gender distribution (% female)	65.8	52.2
*T88.6* **Anaphylactic shock**	89	1506
Average age	56.9	58.5
Gender distribution (% female)	51.7	57.6
*T88.7* **Unspecified adverse effect**	1047	550
Average age	60.9	53.6
Gender distribution (% female)	60.2	51.3

**Pos.** denotes patients which are ADE *positive*, whereas **Neg.** corresponds to patients that are ADE *negative*. The table includes: the total number of patients in each group, the average age and the gender distribution

The clinical database, collected from Karolinska University Hospital in Stockholm, Sweden, consists of medical records for 1.2 million patients from the Stockholm region during a 7 year period (2009–2015). The database include 1877 unique clinical measurements from laboratory tests. In the database, each clinical laboratory test corresponds, therefore, to a multi-variate feature in each ADE dataset. We considered a collection of 15 ADE datasets, where each dataset contained at most 80% of non-empty entries, i.e., had a sparsity of at least 20%. Table 2 provides an overview of the number of features in each ADE dataset and Table 1 gives an overview of the patient characteristics per dataset regarding average age and gender distribution.

**Table 2:** For each ADE dataset, the number of features included in the learning process with different sparsity requirements

	0.2	0.3	0.5	0.7	0.9	0.95	1.0
*D61.1*	16	21	23	34	72	90	186
*E27.3*	11	12	14	19	42	88	137
*G62.0*	4	11	16	19	40	62	151
*I95.2*	11	13	14	20	30	56	180
*L27.0*	4	12	18	25	33	54	162
*L27.1*	6	11	17	24	35	62	169
*M80.4*	9	11	14	19	42	62	170
*O35.5*	1	2	4	15	24	38	73
*T78.2*	8	9	12	17	29	50	168
*T78.3*	8	9	12	17	27	43	131
*T78.4*	8	9	13	17	29	51	194
*T80.1*	11	13	19	25	33	40	131
*T80.8*	11	14	19	25	33	43	128
*T88.6*	11	12	15	21	33	59	202
*T88.7*	11	12	16	21	33	62	217

In particular, each column corresponds to the maximum percentage of empty time series which is tolerated for a dataset. When *τ*_*sp*_=1.0, all the available features are taken into account, regardless of the percentage of empty sequences; the only requirement for a feature to be selected in the latter case is that it contains at least one non-empty sequence

#### Benchmarked methods

The performance of the proposed multi-variate feature representation framework using the three missing value strategies, i.e., plain, mc, and lr, has been evaluated using the *Random Forest* algorithm (RF, [51]). Since our proposed framework is model agnostic, we note that alternative predictive models can be used. The hyper-parameters for RF have been configured as follows: (i) we set the number of trees to 100, (ii) information gain was used as the split criterion (being consistent with the way random subsequences are evaluated and an s-shapelet is selected), and (iii) the number of features to consider at each decision split was set to the default value $\sqrt {m}$, where *m* is the number of features in the dataset.

As a baseline we used a method referred to as *sequence length* representation or sl [20]. This representation has been shown to be the best single-valued representation for clinical laboratory measurement features in the context of detecting ADEs in EHRs, compared to several other multi-variate feature representation techniques that do not take into account the temporal order of the measurements [20].

#### Alphabet tuning

Following the best practice introduced by Zhao et al. [32], we include three different configurations for the SAX alphabet size (*α*). These are *α*={2,3,5}, where the smallest alphabet size is used to reflect two simple states: *low* or *high*, i.e., simply below or above the mean. To allow for more fine grained representations, both an alphabet size of three and five are used to indicate that values can be contained in different regions of the feature value distribution. Since the best choice of *α* is unknown apriori, we here employ a simple strategy to let the learning algorithm dynamically choose the best alphabet size.

#### Sparsity tolerance

We explored different levels of feature sparsity by introducing a threshold *τ*_*sp*_, which limits the maximum fraction of missing data that a feature can contain in order to be selected (and transformed) for training. For instance, *τ*_*sp*_=0.2 indicates that a feature is accepted for training, if it contains no more than 20% of empty sequence records. More specifically, the higher the sparsity threshold the more features are selected to be transformed and, hence, used by the predictive model, in our case RF. Clearly, when *τ*_*sp*_=1.0, all available features are taken into account, regardless of the percentage of empty sequence records.

We refer to Table 2 for the number of features selected for each ADE dataset and sparsity threshold.

#### Evaluation metrics

Since the datasets employed in this study are imbalanced, we used the *Area Under the ROC Curve* (AUC) [52, 53], which has been shown to be a more appropriate classification performance metric compared to accuracy or classification error, when the data source is imbalanced ([54, 55]). AUC represents a range of trade-offs between *sensitivity* and *specificity*, and since both are invariant to the actual balance between classes in the test set, AUC is not biased towards the majority class. Finally, all the AUC results reported in this paper are obtained by stratified 10-fold cross-validation.

## Results

### Sparsity tolerance of baseline

**Fig. 4:**
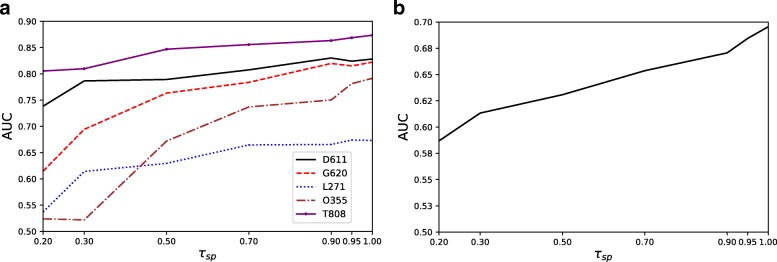
Evaluation of random forest. **a** The AUC obtained by RF on a selection of ADE datasets with increasing sparsity threshold *τ*_*sp*_. Evaluation of random forest. **b** The average AUC obtained by RF on all of the 15 datasets with increasing sparsity threshold *τ*_*sp*_

We first investigate how an effective single-valued feature representation, such as sl, can be affected in terms of predictive performance in the presence of different levels of sparse multi-variate features. Since sl is using the length of the multi-variate feature value (i.e., the length of the sequence assigned in a feature record) as its single-valued representation, empty sequences will be replaced by *∅*. Figure 4a depicts the AUC obtained by RF on a selection of 5 ADE datasets, while Fig. 4b shows the average AUC on all 15 datasets, while increasing the value of *τ*_*sp*_. We observe that predictive performance in terms of AUC increases as sparser features are included in the learning process. Note that although we only show five datasets in Fig. 4a, the performance is similar for the remaining datasets, as it can be confirmed in Table 5.

### Sparsity tolerance of plain, mc, and lr

Next, we investigate whether the consideration of the temporal information provided by the multi-variate features can further improve predictive performance for ADE detection. Moreover, we study whether any further improvements are achieved when including very sparse features, and we explore the most efficient way of representing empty multi-variate feature records. To carry out these experiments, we train an RF by using the three proposed feature representations, namely plain, mc and lr, and measure their respective AUCs for each ADE dataset and for different sparsity levels.

Our findings are depicted in Table 5, where each row refers to one of the 15 ADE datasets, columns correspond to increasing values of *τ*_*sp*_, and each cell reports the average AUC of the corresponding model, obtained by stratified 10-fold cross-validation. For each dataset, the best result is marked in bold. It can be clearly seen how predictive performance in terms of AUC increases as sparser columns are included in the learning process. Indeed, the column corresponding to the best performance is that related to the maximum sparsity threshold, namely *τ*_*sp*_=1.

### Overall comparison

Comparing the performance of all four methods (including sl) at each sparsity level – the highest AUC reached for a given threshold is underlined – we can see how plain and lr are the most effective feature representations, followed by sl. In particular, lr outperforms the other strategies (in terms of number of best results obtained on all of the 15 ADE datasets) for *τ*_*sp*_ equal to 0.2, 0.7, 0.95, and 1, while plain is the best method for *τ*_*sp*_=0.3. For the other two sparsity levels the two methods perform equally well. When considering the best overall result on each ADE dataset, lr reaches the highest AUC on 8 out of 15 cases.

More importantly, one of our main claims is that lr is the strategy which best takes into account the information provided by empty multi-variate feature records. To further prove this claim, we compare the performance of lr against that of plain and sl. Table 3 shows the outcome of comparing lr, plain, and sl over all datasets and sparsity levels. For each value of the sparsity threshold, we report the method achieving the highest AUC on most ADEs, while the number of ADEs where the method is performing best is indicated in brackets. The last column of Table 3 refers to the number of best AUCs obtained by the methods on all of the ADE datasets regardless of the sparsity threshold. Inspecting Table 3, we can notice that lr is the most accurate feature representation strategy for almost every sparsity threshold.

**Table 3:** Comparison between lr and plain (first row) and between lr and sl (second row), for different values of the sparsity threshold

	0.2	0.3	0.5	0.7	0.9	0.95	1.0	Overall
lr vs plain	lr (10)	plain (8)	plain (8)	lr (11)*	lr(11)	lr(9)	lr(10)*	lr (9)
lr vs sl	lr (13)*	lr (14)*	lr (13)*	lr (10)*	lr (13)*	lr (10)	lr(9)	lr (12)*

Each cell of the table reports the method achieving the highest number of best performances (in brackets) among all of the ADE datasets for a particular sparsity level. The last column refers to the number of best AUCs obtained on the ADE datasets regardless of *τ*_*sp*_. An asterisk marks those cases which are proved to be statistically significant within a confidence interval of 0.05

### Statistical significance

Furthermore, we provide a statistical analysis of the previous experiment. Following Demvsar et al. [56] regarding the case of individual comparisons between methods on different datasets, we use the *Wilcoxon signed-rank test* [57] for rejecting the null-hypothesis that the compared methods perform equally well. Those entries of Table 3 where the null-hypothesis is rejected within a confidence interval of 0.05 are marked with an asterisk. In the first comparison, lr performs statistically better than plain when *τ*_*sp*_=0.7,1.0, with a *p*-value of *p*<0.05 in both cases. Conversely, the two cases in which plain outperforms lr are not statistically significant. Concerning the comparison between lr and sl, the null-hypothesis is rejected for *τ*_*sp*_=0.2,0.3,0.5,0.9 (with *p*<0.01), and *τ*_*sp*_=0.7 (with *p*<0.05). Finally, lr proves to be statistically better (*p*<0.05) than sl also when considering the overall best performance on each ADE dataset, as it can be noticed from the last column of Table 3.

### Utility of s-shapelets selected by lr vs. plain

**Fig. 5:**
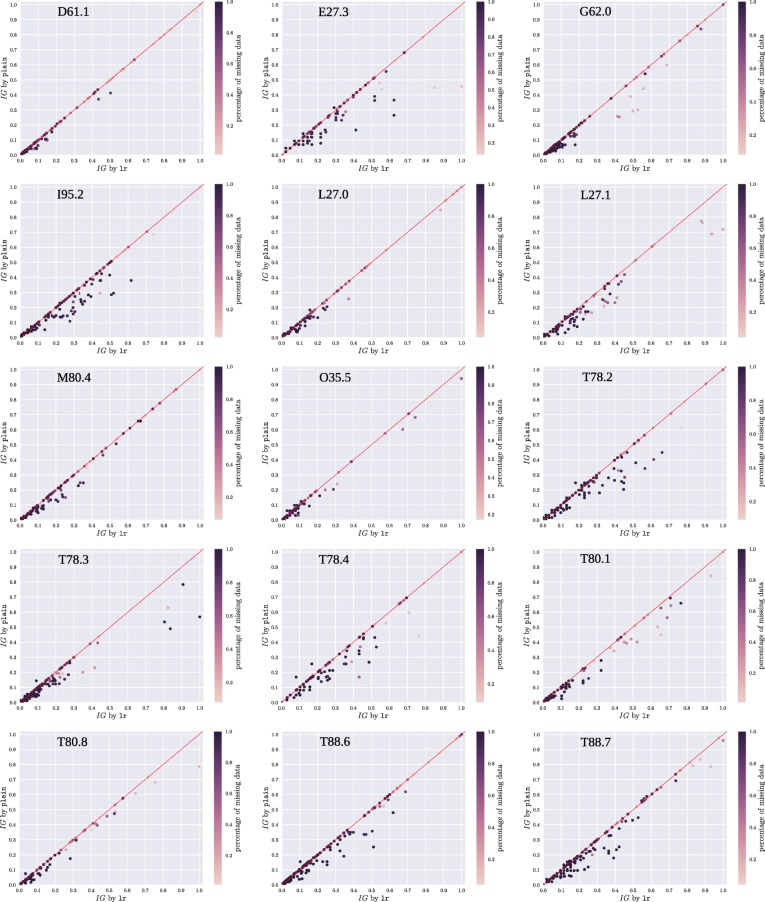
Comparison of plain and lr. The information gain of plain vs lr for all 15 ADE datasets. Overall, we can see that lr captures more informative s-shapelets, especially as the sparsity of the multi-variate representation increases

Our aim in this section is to explore the differences with respect to the utility values obtained for the selected s-shapelets between the strategy that considers sparsity, i.e., lr, and the strategy that does not, i.e., plain. Figure 5, shows the s-shapelet utility, according to information gain, of the s-shapelets that have been generated by using either lr or plain, for all datasets. In fact, the horizontal axes of the subfigures show the information gain for the s-shapelets as computed by lr, while the vertical axes report the information gain computed by plain. The color intensity of the point representing each s-shapelet indicates the sparsity of the multi-variate feature from which it was selected.

By inspecting Fig. 5, we can clearly see how lr is consistently able to select s-shapelets with a higher information gain compared to plain. Also, interestingly, we can identify ADE cases, where the above holds for extremely sparse features, such as *T78.3*, *T80.8*, *T88.7*, *T80.1*, *L27.1*, and *G62.0*. As confirmed by our results, this information gain difference between the two strategies results in a model with higher classification performance.

### Investigation of three ADEs

We further investigated the top-5 features (i.e., features with the highest utility) for lr and plain for three ADEs: *E27.3*, *L27.1*, and *G62.0*. These adverse effects were chosen since they are relatively frequent and the information gain between the baseline and our proposed method differed the most, i.e., there is a conflicting explanation between the two models.

These top-5 features are presented in Table 4. For *E27.3* (i.e., adrenocortical insufficiency), which refers to inefficiency of the hormones cortisol and aldosterone, the lack of these two hormones can cause the body to be unable maintain essential life functions. One cause of this condition is the use of steroids [58], such as *Dehydroepiandro-sterone sulfate*, which is the top-1 variable with consistently elevated values in the blood, found by lr. More importantly, it is well-known in clinical pharmacology that the lack of aldosterone can cause persistently low or uncontrolled levels of sodium, potassium, and cortisol in the blood [58]. This has also been confirmed by the clinical pharmacologists involved in our study. Interestingly, both sodium and potassium are included in the list of top-5 features identified by lr. On the other hand, plain manages to identify rather obvious features, such as reduced levels of cortisol and hemoglobin, which are typically present in the occurrence of adrenal hemorrhage [58].

**Table 4:** The most important multi-variate features used by the transformation framework for three of the datasets (from left, *E27.3*, *L27.1* and *G62.0*)

	Adrenocortical insufficiency		Localized skin eruption		Polyneuropathy	
	lr	plain	lr	plain	lr	plain
1	Dehydroepiandro-sterone sulfate	Lymphocytes	Erythrocytes	MCHC	Erythrocytes	MCHC
2	Potassium ion	Cortisol	MCHC	Erythrocytes	MCHC	Erythrocytes
3	Neutrophilocytes	Hemoglobin	Calcium	Calcium	MCH	MCH
4	Lymphocytes	Sedimentation reaction	Bilirubins	Creatininium	Bilirubins	Calcium
5	Sodium ion	Erythrocytes	Creatininium	Carbamide	Calcium	Creatininium

Regarding *L27.1* (i.e., localized skin eruption), both methods mostly agree on the most informative features, which include high levels of erythrocytes and increased bilirubin levels in the liver. Both are reported as signs of drug-induced skin disorder, with the second one also indicating anemia in the blood, which is a typical cause of localized skin eruption [59].

Finally, polyneuropathy, which refers to the damage of peripheral nerves can be medication induced. As shown in Table 4, calcium is a consistent predictor for both plain and lr. This also abides to the findings of Fernybough and Calcutt [60] that reduced or irregular levels of calcium can indicate peripheral neuropathy. More importantly, patients with peripheral neuropathy typically exhibit elevated erythrocyte sedimentation rate in their blood [61], which is also consistent with our findings.

In summary, it is clear that both plain and lr manage to identify important features that are connected with the corresponding ADEs. Based on the existing literature and our consultation with our collaborating clinical pharmacologists, our findings presented in Table 4 are not substantially significant as they are already known to clinical pharmacologists. Nonetheless, they demonstrate that our proposed method works in practice, while lr is shown to be highly robust to sparse temporal features without compromising predictive performance.

## Discussion

The classical approach for dealing with time evolving representations of medical records is to either summarize the data using simple features [17–19] or to employ population-based feature extraction [30, 31]. However, these summarization heuristics compromise the quality of the feature space by either ignoring the temporal dimension of the data or by simplifying it. The closest related work is the *random dynamic subsequence* method [32], which is included in the experimental evaluation. The results of our empirical evaluation demonstrate that the *random dynamic subsequence* approach suffers from two main weaknesses: (1) the chosen distance function, i.e., Levenshtein distance, is highly dependent on the sequence length and (2) it cannot effectively handle and exploit the high degree of sparsity in the feature space. To overcome these limitations, we first extend the method to allow for different distance functions and compute these on equi-length representations, and secondly, we introduce three novel strategies for inferring the importance of values missing not at random. As such, our findings in this study advance the current state-of-the-art in terms of detecting ADEs from time-evolving and sparsely recorded medical record systems.

More concretely, we observed how predictive performance in terms of AUC increases as sparser features are included in the learning process when employing the baseline method, sl, which simply employs the number of recordings (length) per clinical laboratory measurement as a feature for the ADE prediction task. This suggests that the presence or absence, as well as the number of times a clinical laboratory measurement has been taken for a patient can constitute a promising ADE predictor.

Moreover, as far as the three strategies for handling multi-variate feature sparsity are concerned, our findings suggest that the proposed techniques provide good trade-offs between feature sparsity and predictive performance in terms of AUC. Consequently, this indicates that strategies that take into account the sparsity of the data typically outperform those that do not. Hence, this study demonstrates that temporal multi-variate EHR features of variable length and with high levels of sparsity can be effectively utilized to predict ADEs.

Furthermore, special attention has to be given on how to encode empty feature records in the transformed dataset when evaluating the utility of candidate s-shapelet. Indeed, the s-shapelet interpretation discussed above can easily become infeasible when the binary split introduced by the s-shapelet is affected by a large amount of empty sequences. For example, consider the feature representation of sl. According to this strategy, *∅*s are replaced with 0, regardless of the class distribution. The same holds for plain and mc, which map *∅* with |*s*| and the most common observed distance value, respectively. More generally, a *static* encoding of missing data, ignoring the information given by class distribution, affects the quality of an s-shapelet. Conversely, by employing a strategy such as lr, the choice of the encoding of empty sequences *dynamically* fits the class balance.

One limitation of the current study, is that, while the framework is step agnostic, a single configuration is evaluated in the experiments. As such, there might be configurations that provide further improvements in terms predictive performance against the state-of-the-art. We plan to explore this in future studies. Moreover, the novel framework is only evaluated on a single task (albeit using many instantiations of different datasets and targets). As such, it is rather difficult to generalize the results outside the scope of the current study, i.e., to detect other medical conditions except for ADEs. However, due to the fact that the results are consistent between different datasets, the relative performance of the investigated strategies are not expected to differ, only the absolute performance.

From a clinical perspective we acknowledge the limitations of the quality of our data sets pertaining to the nature in which ADEs are underreported by clinicians. ADEs are often not the primary reason for clinical encounters and can be seldom investigated or recognized by clinicians with a high degree of certainty. For this reason our negative set may indeed contain a high proportion of undetected and unreported ADE cases which could be detrimental to classification performance results. Nevertheless, we emphasize that our classification results demonstrate a clear ability to differentiate reported ADE cases from similar non-ADE cases or cases where the relevant ADE was overlooked during a clinical encounter.

Additionally, for future studies we would wish to exploit, not only laboratory test data, but medication data due to the high clinical relevancy of this information for improving classification and for the potential of uncovering relations between certain medications and ADEs. For the purposes of the current study, laboratory test data was utilized as it’s sequential numerical nature was most relevant for the approach of our framework which involves summarizing numerical medical information for application with traditional machine learning algorithms. However, the uncovering of medication-based insights could indeed validate our approach by discovering known relations between ADEs and certain medications while also generating novel hypotheses for medications which contribute to ADEs under particular conditions.

## Conclusions

We have proposed a novel three-phase symbolic transformation framework for classification of complex and sparse temporal multi-variate feature datasets. These types of complex features are common in EHRs and can be effectively used for various prediction tasks, such as ADE detection. Moreover, applying machine learning algorithms to learn models from such data is typically challenging, due to the variable length and the sparsity of such features. In this study, we proposed and formalized a way of handling these types of multi-variate and sparse features, with focus on ADE prediction from EHRs.

Moreover, our experimental analysis demonstrated the importance of including temporal information in the multi-variate feature representation, and emphasized the usefulness of such information when the data features are extremely sparse. It is worthwhile to note that a possibly useful empty feature record, although being static, can add utility to a feature. Moreover, we should mention that when temporal information is taken into account during the transformation process, special attention has to be taken when encoding and handling empty sequences. The results in Table 5 confirm that different treatments of sparse datasets in a time dependent scenario result in different impact on the AUC of the resulting model.

**Table 5:** AUC obtained by RF on 15 ADE datasets: 4 methods are compared, respectively sl, plain, mc and lr

		0.2	0.3	0.5	0.7	0.9	0.95	1.0
**D61.1**	plain	78.151	80.842	80.817	80.909	84.107	**84.801**	82.484
	mc	76.876	78.132	80.163	78.854	81.259	82.946	81.720
	lr	77.978	80.720	79.961	79.675	83.624	83.610	81.510
	sl	73.846	78.653	78.928	80.756	83.016	82.412	82.817
**E27.3**	plain	51.916	58.345	56.302	59.870	58.882	62.518	66.854
	mc	48.953	50.052	46.695	50.126	61.773	59.444	58.854
	lr	58.445	58.637	56.816	61.179	59.170	64.162	**67.266**
	sl	60.763	62.054	59.670	58.718	58.035	64.686	66.500
**G62.0**	plain	64.443	71.325	75.452	77.247	78.839	79.074	80.279
	mc	66.228	72.700	71.376	76.974	74.769	74.146	74.713
	lr	64.713	70.756	74.787	77.169	78.992	79.357	79.418
	sl	61.473	69.429	76.335	78.379	81.980	81.509	**82.222**
**I95.2**	plain	56.157	57.338	54.871	52.257	54.046	**59.855**	56.369
	mc	57.486	57.390	54.750	49.333	53.480	51.742	57.391
	lr	58.052	54.797	58.165	53.184	56.145	57.145	56.362
	sl	47.671	49.289	51.080	51.509	53.065	54.267	53.943
**L27.0**	plain	60.374	68.063	66.689	67.815	65.273	67.416	65.322
	mc	56.065	62.683	66.766	65.527	67.431	66.443	64.500
	lr	56.585	66.422	66.256	67.020	66.277	**68.424**	66.110
	sl	55.400	62.981	61.919	64.648	64.597	65.136	68.271
**L27.1**	plain	58.130	61.600	63.471	64.835	63.012	62.454	61.798
	mc	56.093	55.029	67.179	61.970	64.556	61.222	62.455
	lr	58.636	64.100	65.983	66.264	67.196	63.884	64.012
	sl	53.709	61.418	62.955	66.461	66.554	**67.397**	67.316
**M80.4**	plain	55.646	54.745	59.375	59.029	68.713	67.004	65.396
	mc	55.729	55.770	52.641	58.996	66.863	63.000	63.507
	lr	55.753	58.056	58.849	59.760	68.912	**69.119**	67.756
	sl	47.933	57.149	52.678	58.414	67.945	65.199	64.845
**O35.5**	plain	51.650	52.557	64.963	73.161	77.383	78.590	80.298
	mc	52.741	52.962	55.361	68.843	70.114	72.079	70.104
	lr	52.756	53.551	68.318	76.971	78.332	80.322	**81.324**
	sl	52.401	52.188	67.201	73.696	75.025	78.140	79.142
**T78.2**	plain	53.028	51.573	56.977	56.538	57.153	58.709	59.229
	mc	55.811	54.349	53.146	55.771	53.260	54.417	53.092
	lr	52.924	52.369	55.286	59.569	**63.066**	57.775	59.254
	sl	53.113	50.471	50.492	57.561	56.850	58.646	59.421
**T78.3**	plain	51.177	54.073	52.614	54.575	58.973	59.449	63.647
	mc	48.308	50.692	51.378	53.746	56.715	55.334	58.111
	lr	51.762	53.238	55.155	56.952	59.213	61.924	**66.209**
	sl	50.514	51.851	54.273	53.531	56.337	59.759	64.728
**T78.4**	plain	51.937	54.675	58.151	56.976	57.124	56.506	**59.162**
	mc	52.176	55.459	55.644	54.720	53.475	55.145	56.218
	lr	54.434	58.122	54.655	57.019	56.627	58.496	58.549
	sl	49.903	49.876	47.081	50.235	54.326	56.986	58.444
**T80.1**	plain	78.962	77.270	84.589	83.097	**86.832**	85.994	84.189
	mc	73.207	72.912	73.492	78.945	74.963	79.334	74.592
	lr	78.930	80.478	83.233	83.739	84.677	84.879	85.803
	sl	75.125	76.542	79.956	81.759	81.413	83.918	82.726
**T80.8**	plain	80.744	81.769	83.486	83.704	85.648	86.136	86.751
	mc	71.968	71.717	76.488	77.570	78.308	78.182	80.725
	lr	81.119	81.211	85.431	85.210	86.319	86.715	86.627
	sl	80.537	80.973	84.689	85.569	86.332	86.875	**87.351**
**T88.6**	plain	61.998	60.731	62.299	62.830	65.113	63.499	62.249
	mc	58.342	58.993	63.476	57.263	59.652	59.568	61.812
	lr	60.283	60.159	62.019	63.256	60.864	61.744	**64.120**
	sl	57.623	57.248	57.450	57.417	58.190	57.672	60.403
**T88.7**	plain	58.971	60.742	62.399	62.239	62.552	63.803	65.352
	mc	56.875	57.931	62.472	60.892	63.013	62.435	64.209
	lr	60.580	60.462	62.803	61.543	62.588	64.137	**66.276**
	sl	60.297	59.862	61.498	61.811	62.259	64.001	65.139

For each ADE, several sparsity threshold are considered, from 0.2 up to 1.0. The AUC reported for each combination is averaged by cross-validation on 10 different dataset splits. The best result is bold, and the highest AUC for each threshold underlined

Finally, an advantage of the proposed framework is that its three phases are method agnostic, i.e., the framework allows for exchanging the sub-tasks of each phase. For instance, one could use different machine learning models, different normalization techniques, symbolic approximations, or distance measures. As such, the proposed framework provides an important building block for future exploitation with one important avenue for future work being the evaluation into alternative choices which may affect the predictive performance of the learned representations.

## Additional file

Additional file 1 Top 20 least sparse features. The top 20 least sparse multi-variate features per dataset and patient group (i.e., ADE positive and ADE negative patients). The feature names are encoded as Nomenclature, Properties and Units terminology codes and the files include short definitions of these names. (XLS 69 kb)

## Abbreviations

AUC: Area under receiver operator curve
ADE: Adverse drug event
ATC: Anatomical therapeutic chemical classification
EHR: Electronic health record
DFT: Discrete Fourier transformation
DWT: Discrete wavelet transformation
ICD: International classification of disease
MNAR: Missing not-at-random
NPU: Nomenclature, Properties and Units codes
PAA: Piecewise aggregate approximation
ROC: Receiver operator curve
RF: Random forest
SAX: Symbolic aggregate approximation
